# Activity of ceftolozane/tazobactam against Gram-negative isolates from patients with lower respiratory tract infections – SMART United States 2018–2019

**DOI:** 10.1186/s12866-021-02135-z

**Published:** 2021-03-06

**Authors:** James A. Karlowsky, Sibylle H. Lob, Katherine Young, Mary R. Motyl, Daniel F. Sahm

**Affiliations:** 1 IHMA, 2122 Palmer Drive, Schaumburg, IL 60173 USA; 2 Department of Medical Microbiology and Infectious Diseases, Max Rady College of Medicine, University of Manitoba, Winnipeg, MB R3E 0J9 Canada; 3 Merck & Co., Inc., Kenilworth, NJ 07033 USA

**Keywords:** Ceftolozane/tazobactam, Enterobacterales, *Pseudomonas aeruginosa*, United states, SMART, Surveillance

## Abstract

**Background:**

Ceftolozane/tazobactam (C/T) is approved in 70 countries, including the United States, for the treatment of patients with hospital-acquired and ventilator-associated bacterial pneumonia caused by susceptible Gram-negative pathogens. C/T is of particular importance as an agent for the treatment of multidrug-resistant (MDR) *Pseudomonas aeruginosa* infections. The current study summarizes 2018–2019 data from the United States on lower respiratory tract isolates of Gram-negative bacilli from the SMART global surveillance program. The CLSI reference broth microdilution method was used to determine in vitro susceptibility of C/T and comparators against isolates of *P. aeruginosa* and Enterobacterales.

**Results:**

C/T inhibited 96.0% of *P. aeruginosa* (*n* = 1237) at its susceptible MIC breakpoint (≤4 μg/ml), including > 85% of meropenem-nonsusceptible and piperacillin/tazobactam (P/T)-nonsusceptible isolates and 76.2% of MDR isolates. Comparator agents demonstrated lower activity than C/T against *P. aeruginosa*: meropenem (74.8% susceptible), cefepime (79.2%), ceftazidime (78.5%), P/T (74.4%), and levofloxacin (63.1%). C/T was equally active against ICU (96.0% susceptible) and non-ICU (96.7%) isolates of *P. aeruginosa*. C/T inhibited 91.8% of Enterobacterales (*n* = 1938) at its susceptible MIC breakpoint (≤2 μg/ml); 89.5% of isolates were susceptible to cefepime and 88.0% susceptible to P/T. 67.1 and 86.5% of extended-spectrum β-lactamase (ESBL) screen-positive isolates of *Klebsiella pneumoniae* (*n* = 85) and *Escherichia coli* (*n* = 74) and 49.6% of MDR Enterobacterales were susceptible to C/T. C/T was equally active against ICU (91.3% susceptible) and non-ICU (92.6%) Enterobacterales isolates.

**Conclusion:**

Data from the current study support the use of C/T as an important treatment option for lower respiratory tract infections including those caused by MDR *P. aeruginosa*.

**Supplementary Information:**

The online version contains supplementary material available at 10.1186/s12866-021-02135-z.

## Background

Lower respiratory tract infections are common in hospitalized patients, particularly among patients in intensive care units (ICUs), and are associated with significant morbidity and mortality [1]. Gram-negative bacilli, including *Pseudomonas aeruginosa* and Enterobacterales are frequent and important pathogens in patients with hospital-acquired and ventilator-associated pneumonia. *P. aeruginosa* is a particularly important nosocomial pathogen because it possesses intrinsic resistance to several antimicrobial agent classes and not uncommonly demonstrates resistance to antipseudomonal agents.

Ceftolozane/tazobactam (C/T) is a parenteral agent that combines an antipseudomonal cephalosporin with tazobactam, an established β-lactamase inhibitor of many Ambler class A β-lactamases (excluding KPC-type and some GES-type carbapenemases and the ESBL PER-1) [2]. C/T demonstrates potent in vitro activity against many clinically important Gram-negative bacilli and enhanced activity against *P. aeruginosa* [3–9]. C/T is approved in 70 countries, including the United States for the treatment of patients with hospital-acquired and ventilator-associated bacterial pneumonia (3 g every 8 h), and in 75 countries for the treatment of complicated intraabdominal infection (1.5 g every 8 h, used in combination with metronidazole), and complicated urinary tract infection (1.5 g every 8 h), including pyelonephritis [10]. C/T is an important agent for the treatment of patients with severe infections due to multidrug-resistant (MDR) *P. aeruginosa* [11], which is a common phenotype in this pathogen in many countries, including the United States [4, 6, 12–14]. C/T may also provide an alternative to carbapenems for the treatment of infections caused by Gram-negative bacilli, including extended-spectrum β-lactamase (ESBL)-producing isolates [15–17].

The current report summarizes antimicrobial susceptibility testing results for C/T for isolates of *P. aeruginosa* and Enterobacterales submitted to the SMART (Study for Monitoring Antimicrobial Resistance Trends) global surveillance program in 2018–2019 by clinical laboratories from the United States, focusing on isolates from lower respiratory tract infections.

## Results

Isolates of *P. aeruginosa* were highly susceptible (96.0%) to C/T (MIC ≤4 μg/ml) (Table 1); < 80% of isolates of *P. aeruginosa* were susceptible to the other β-lactams tested, including cefepime, ceftazidime, piperacillin/tazobactam (P/T), and meropenem. Susceptibility to amikacin (96.0%) was identical to C/T against *P. aeruginosa*. Most isolates of Enterobacterales (91.8%) were also susceptible to C/T (MIC ≤2 μg/ml) (Table 1). The susceptibility of isolates of Enterobacterales to C/T was 2.3 (cefepime) to 11.7 (ceftriaxone) percentage points higher than all other β-lactams tested except meropenem (98.0% susceptible). Amikacin was the most active agent tested against Enterobacterales (98.8% susceptible). Against individual species of Enterobacterales, C/T was most active against *Proteus mirabilis* (99.2% susceptible), *Klebsiella oxytoca* (97.8%), and *Escherichia coli* (97.2%). The study identified 49 (4.0%) isolates of *P. aeruginosa* and 158 (8.2%) isolates of Enterobacterales (Table 1, Additional file 1: Table S1) that were C/T-nonsusceptible; 70% (110/158) of C/T-nonsusceptible Enterobacterales isolates were intrinsic AmpC producers. Table 2 summarizes the in vitro activities of C/T and comparator agents against nonsusceptible subsets of isolates and MDR isolates of *P. aeruginosa* and Enterobacterales; 14.6% (181/1237) of *P. aeruginosa* and 12.3% (238/1938) of Enterobacterales possessed MDR phenotypes. Table S2 shows the 5 most commonly identified MDR phenotypes among *P. aeruginosa* and Enterobacterales (Additional file 2). C/T inhibited 76.2% of all MDR isolates of *P. aeruginosa* (including 96.8% of isolates of the most common phenotype: resistant to aztreonam, imipenem, and levofloxacin [*n* = 31], Additional file 2: Table S2), and > 80% of cefepime-, ceftazidime-, meropenem-, or P/T-nonsusceptible *P. aeruginosa* (Table 2). The susceptibility of MDR isolates of *P. aeruginosa* to C/T was 58.5–72.3 percentage points higher than to other β-lactams and levofloxacin. Only amikacin showed a higher rate of susceptibility than C/T against MDR isolates of *P. aeruginosa*. C/T inhibited 49.6% of MDR Enterobacterales (including 90.4% of isolates of the most common phenotype: resistant to aztreonam, cefepime, ceftazidime, and levofloxacin [*n* = 52], Additional file 2: Table S2), and > 50% of cefepime- or ceftazidime-nonsusceptible Enterobacterales (Table 2). The susceptibility of MDR isolates of Enterobacterales to C/T was 6.7 (P/T) to 42.4 (ceftriaxone) percentage points higher than to non-carbapenem β-lactam comparator antimicrobial agents and levofloxacin. Only amikacin and carbapenems showed higher susceptibility rates than C/T against MDR isolates of Enterobacterales. 67.1 and 86.5% of ESBL screen-positive isolates of *Klebsiella pneumoniae* (*n* = 85) and *Escherichia coli* (*n* = 74) were susceptible to C/T compared to 52.9 and 79.7%, respectively, for P/T.

**Table 1:** Susceptibility of lower respiratory tract infection isolates of *P. aeruginosa* and Enterobacterales to C/T and comparator agents^a,b^ – SMART 2018–2019, United States

Species	*n*	% Susceptible									
		C/T	P/T	FEP	CAZ	CRO	ATM	MEM	IMI	LVX	AMK
*P. aeruginosa*	1237	96.0	74.4	79.2	78.5	NA	68.2	74.8	65.2	63.1	96.0
All Enterobacterales^c^	1938	91.8	88.0	89.5	83.4	80.1	84.2	98.0	86.4	82.2	98.8
*K. pneumoniae*	432	92.6	86.8	82.2	79.9	80.3	81.9	96.5	95.1	81.0	98.4
*E. coli*	359	97.2	91.9	84.7	82.2	79.4	82.5	99.7	99.7	66.6	98.9
*S. marcescens*	261	95.8	93.5	96.6	95.8	84.3	93.5	96.9	84.3	90.8	97.3
*E. cloacae*	176	77.8	79.0	89.8	72.2	69.3	73.9	98.9	94.3	94.3	100
*K. aerogenes*	152	79.6	73.0	94.1	70.4	67.8	72.4	98.0	73.0	93.4	100
*K. oxytoca*	138	97.8	87.7	94.9	96.4	87.0	91.3	97.8	97.1	99.3	100
*P. mirabilis*	121	99.2	100	95.0	96.7	95.9	97.5	100	19.8	61.2	99.2
*P. aeruginosa* + Enterobacterales	3175	93.5	82.7	85.5	81.5	NA	78.0	88.9	78.2	74.8	97.7

^a^ Abbreviations: C/T, ceftolozane/tazobactam; P/T, piperacillin/tazobactam; FEP, cefepime; CAZ, ceftazidime; CRO, ceftriaxone; ATM, aztreonam; MEM, meropenem; IMI, impenem; LVX, levofloxacin; AMK, amikacin, NA, not available

^b^ Table does not show results for colistin because *P. aeruginosa* and Enterobacterales cannot be categorized as susceptible to colistin using CLSI M100 (2020) MIC breakpoint criteria

^c^ Table only shows data for individual species of Enterobacterales when *n* > 100

**Table 2:** Susceptibility of phenotypic subsets of lower respiratory tract infection isolates of *P. aeruginosa* and Enterobacterales to C/T and comparator agents^a,b^ – SMART 2018–2019, United States

Species/phenotype	*n*	% Susceptible									
		C/T	P/T	FEP	CAZ	CRO	ATM	MEM	IMI	LVX	AMK
*P. aeruginosa*											
FEP-nonsusceptible	257	82.1	12.5	0	18.3	NA	9.3	38.9	35.4	31.1	86.4
CAZ-nonsusceptible	266	81.6	10.2	21.1	0	NA	12.8	46.6	41.0	38.4	88.7
MEM-nonsusceptible	312	87.8	41.7	49.7	54.5	NA	31.1	0	7.1	30.1	90.1
P/T-nonsusceptible	317	86.8	0	29.0	24.6	NA	12.3	42.6	40.4	36.0	91.2
MDR	181	76.2	8.8	12.7	17.1	NA	3.9	17.7	15.5	17.7	82.3
Enterobacterales											
FEP-nonsusceptible	204	59.3	50.5	0	10.3	2.0	9.3	84.8	78.4	31.4	91.7
CAZ-nonsusceptible	321	52.7	43.9	43.0	0	3.7	11.2	89.4	81.9	52.0	95.0
MEM-nonsusceptible	39	12.8	10.3	20.5	12.8	7.7	7.7	0	0	38.5	84.6
P/T-nonsusceptible	232	36.2	0	56.5	22.4	16.4	22.8	84.9	80.2	66.4	94.0
MDR	238	49.6	42.9	30.7	8.8	7.1	9.2	84.9	73.1	36.6	92.4
*K. pneumoniae*^c^											
FEP-nonsusceptible	77	63.6	49.4	0	3.9	2.6	5.2	80.5	79.2	28.6	90.9
CAZ-nonsusceptible	87	63.2	47.1	14.9	0	8.1	11.5	82.8	80.5	33.3	92.0
P/T-nonsusceptible	57	45.6	0	31.6	19.3	29.8	31.6	73.7	70.2	47.4	87.7
ESBL screen-positive^d^	85	67.1	52.9	11.8	5.9	0	9.4	82.4	80.0	32.9	91.8
MDR	66	59.1	45.5	1.5	0	0	0	77.3	75.8	25.8	89.4
*E. coli*^c^											
FEP-nonsusceptible	55	87.3	80.0	0	12.7	0	12.7	98.2	98.2	14.6	92.7
CAZ-nonsusceptible	64	84.4	75.0	25.0	0	3.1	6.3	98.4	98.4	29.7	95.3
P/T-nonsusceptible	29	69.0	0	62.1	44.8	48.3	51.7	96.6	96.6	51.7	93.1
ESBL screen-positive^d^	74	86.5	79.7	25.7	16.2	0	16.2	98.7	98.7	25.7	94.6
MDR	45	82.2	73.3	4.4	0	0	2.2	97.8	97.8	11.1	93.3
*P. aeruginosa* + Enterobacterales											
FEP-nonsusceptible	461	72.0	29.3	0	14.7	NA	9.3	59.2	54.4	31.2	88.7
CAZ-nonsusceptible	587	65.8	28.6	33.0	0	NA	11.9	70.0	63.4	45.8	92.2
MEM-nonsusceptible	351	79.5	38.2	46.4	49.9	NA	28.5	0	6.3	31.1	89.5
P/T-nonsusceptible	549	65.4	0	40.6	23.7	NA	16.8	60.5	57.2	48.8	92.4
MDR	419	61.1	28.2	22.9	12.4	NA	6.9	55.8	48.2	28.4	88.1

^a^ Abbreviations: C/T, ceftolozane/tazobactam; P/T, piperacillin/tazobactam; FEP, cefepime; CAZ, ceftazidime; CRO, ceftriaxone; ATM, aztreonam; MEM, meropenem; IMI, impenem; LVX, levofloxacin; AMK, amikacin; NA, not available

^b^ Table does not show results for colistin because *P. aeruginosa* and Enterobacterales cannot be categorized as susceptible to colistin using CLSI M100 (2020) MIC breakpoint criteria

^c^ MEM-nonsusceptible *E. coli* (*n* = 1) and MEM- nonsusceptible *K. pneumoniae* (*n* = 15) were not included in the table because there were too few isolates

^d^ ESBL screen-positive isolates of *K. pneumoniae* and *E. coli* were identified by a CRO-nonsusceptible phenotype

Table 3 summarizes the species distribution among Enterobacterales collected from patients with lower respiratory tract infections in ICU and non-ICU wards and shows very similar species distributions between the two ward types. Table 4 shows the susceptibility to various antimicrobial agents of *P. aeruginosa* and Enterobacterales from patients with lower respiratory tract infections in ICU and non-ICU wards. Percent susceptible rates were significantly lower for P/T, cefepime, ceftazidime, and meropenem for ICU ward isolates compared to non-ICU ward isolates for *P. aeruginosa* (*P* < 0.05); no significant differences between ICU and non-ICU ward isolates were identified for any agent tested against Enterobacterales (*P* > 0.05). C/T was equally active against ICU and non-ICU isolates of *P. aeruginosa* (96.0 and 96.7% susceptible, respectively) and of Enterobacterales (91.3 and 92.6%, respectively).

**Table 3:** Species distribution among Enterobacterales collected from patients with RTI in ICU (*n* = 1005) and non-ICU wards (*n* = 726) – SMART 2018–2019, United States^a^

Species of Enterobacterales	ICU, *n* (% of ICU isolates of Enterobacterales)	Non-ICU, *n* (% of non-ICU isolates of Enterobacterales)
*K. pneumoniae*	234 (23.3)	156 (21.5)
*E. coli*	187 (18.6)	129 (17.8)
*S. marcescens*	130 (12.9)	102 (14.0)
*E. cloacae*	86 (8.6)	66 (9.1)
*K. aerogenes*	84 (8.4)	52 (7.2)
*K. oxytoca*	71 (7.1)	53 (7.3)
*P. mirabilis*	60 (6.0)	48 (6.6)
Other species	153 (15.2)	120 (16.5)

^a^ Of the 1938 isolates of Enterobacterales, 57 were from emergency rooms and 150 did not have a location provided and were excluded from this table

**Table 4:** Susceptibility of ICU and non-ICU ward subsets of lower respiratory tract infection isolates of *P. aeruginosa* and Enterobacterales to C/T and comparator agents^a,b,c^ – SMART 2018–2019, United States

Species/ward type	*n*	% Susceptible										% MDR
		C/T	P/T	FEP	CAZ	CRO	ATM	MEM	IMI	LVX	AMK	
*P. aeruginosa*^d^												
ICU	495	96.0	**72.3**	**78.0**	**76.6**	NA	68.1	**72.9**	64.4	66.7	96.2	**17.8**
Non-ICU	583	96.7	**79.4**	**83.0**	**82.9**	NA	72.2	**79.8**	69.0	61.9	96.6	**10.5**
Enterobacterales^e^												
ICU	1005	91.3	87.4	89.4	83.2	80.5	83.3	97.5	87.5	81.7	99.0	13.0
Non-ICU	726	92.6	89.3	89.4	83.3	79.6	85.1	98.5	85.1	83.0	98.6	11.7
*P. aeruginosa* + Enterobacterales												
ICU	1500	92.9	82.4	85.6	81.0	NA	78.3	89.4	79.9	76.7	98.1	**14.6**
Non-ICU	1309	94.4	84.9	86.6	83.1	NA	79.4	90.1	77.9	73.6	97.7	**11.2**

^a^ Abbreviations: C/T, ceftolozane/tazobactam; P/T, piperacillin/tazobactam; FEP, cefepime; CAZ, ceftazidime; CRO, ceftriaxone; ATM, aztreonam; MEM, meropenem; IMI, impenem; LVX, levofloxacin; AMK, amikacin; NA, not available

^b^ Table does not show results for colistin because *P. aeruginosa* and Enterobacterales cannot be categorized as susceptible to colistin using CLSI M100 (2020) MIC breakpoint criteria

^c^ Statistically significant differences between ICU and non-ICU are shown in bold font

^d^ Of the 1237 isolates *P. aeruginosa*, 48 were from emergency rooms and 111 did not have a location provided and were excluded from this analysis

^e^ Of the 1938 isolates of Enterobacterales, 57 were from emergency rooms and 150 did not have a location provided and were excluded from this analysis

## Discussion

Empirical regimens to treat hospital-acquired and ventilator-associated pneumonia are based on the most prevalent pathogens causing infection and their antimicrobial susceptibility patterns. Prompt and appropriate antimicrobial treatment decreases infection-related morbidity and mortality; resistant bacteria lead to inadequate empirical treatment and are associated with patient mortality [1]. In the current study of lower respiratory tract infection isolates of Gram-negative bacilli, C/T was active against 96.0% of isolates of *P. aeruginosa,* 91.8% of isolates of Enterobacterales, and 93.5% of *P. aeruginosa* and Enterobacterales combined, which accounted for > 85% of all collected Gram-negative pathogens (Table 1). C/T has sustained its potent in vitro activity against *P. aeruginosa* and Enterobacterales with clinical use since it was first approved by the US FDA in 2014 and by EMA in 2015 [7, 8]. C/T was approved for the treatment of adult patients with hospital-acquired and ventilator-associated bacterial pneumonia by the US FDA and EMA in 2019.

In the current study, 4.0% of isolates of *P. aeruginosa* and 8.2% isolates of Enterobacterales were C/T-nonsusceptible; 70% of the C/T-nonsusceptible Enterobacterales isolates were intrinsic AmpC producers. AmpC induction is an important β-lactam resistance mechanism. Different groups of β-lactams have different abilities to cause derepression of *ampC* [18, 19]. Ceftolozane is an extended-spectrum cephalosporin, and like other extended-spectrum cephalosporins (e.g., ceftazidime) is a poor inducer of *ampC* expression [18–20], making it an attractive treatment option for infections caused by *P. aeruginosa* and Enterobacterales. Ceftolozane is structurally similar to ceftazidime but possesses a pyrazole substituent at the 3-position side chain which provides steric hinderance and prevents or slows hydrolysis by most AmpC β-lactamases. Tazobactam is an irreversible inhibitor of some class C β-lactamases and a number of class A β-lactamases (most ESBLs, not KPCs). Tazobactam is generally a poor inducer of AmpC; however, when high expression levels of AmpC are present, tazobactam may be overwhelmed (similar to clavulanic acid and sulbactam), making it a poor inhibitor of AmpC. This may lead to resistance among Enterobacterales [21].

C/T is highly active against *P. aeruginosa* which contain PDC (*Pseudomonas* derived cephalosporinase) a constitutive Ambler class C β-lactamase (AmpC) with high sequence polymorphism (> 300 sequence variants have been identified) [22, 23]. Recently, rare, individual isolates of *P. aeruginosa* that developed resistance to C/T and ceftazidime/avibactam during therapy by acquisition of structural mutations in, and over-expression of, PDC in a highly derepressed *ampC* background have been reported [24, 25].

Previous studies of *P. aeruginosa* have identified one or more of OprD loss, *ampC* derepression, or hyperexpression of efflux pumps as likely mechanisms leading to P/T, ceftazidime, cefepime, and carbapenem resistance [9, 22]. Isolates of *P. aeruginosa* with carbapenem-resistant phenotypes generally do not carry carbapenemases [22, 26]. OprD porin loss and active efflux appear to have no impact on C/T MICs [9, 22, 24]. C/T is widely regarded as the most suitable option for the treatment of infections caused by non-carbapenemase-producing MDR *P. aeruginosa* [11].

The combination of tazobactam with ceftolozane protects ceftolozane from hydrolysis by the majority of ESBL-producing isolates of *E. coli* and *K. pneumoniae*; ~ 90% of ESBL-positive *E. coli* and ~ 70% of ESBL-positive *K. pneumoniae* are susceptible to C/T [4, 5, 7–9, 17]. Lower MICs in ESBL-producing *E. coli* (MIC_50_/MIC_90_, 0.5/4 μg/ml) than in *K. pneumoniae* (MIC_50_/MIC_90_, 4/> 32 μg/ml) reflect differences in ESBL genes typically present in *E. coli* (*bla*_CTX-M_) and *K. pneumoniae* (*bla*_TEM/SHV_) [4, 5, 7–9]. In the current study, C/T demonstrated greater in vitro activity against all Enterobacterales and against ESBL screen-positive isolates than P/T and currently available advanced-generation cephalosporins, as observed by previous investigators [4, 5, 7–9, 17].

Increased carbapenem use is observed in areas with greater prevalence of ESBL-producing Enterobacterales [15]. Carbapenems are frequently recommended for the treatment of ESBL-producing infections, even when isolates are susceptible in vitro to other β-lactams [15]. This practice as well as fluoroquinolone and ceftazidime use have contributed to the emergence and spread of carbapenemase-producing *Enterobacterales*, for which therapeutic options are limited. Adequately powered randomized controlled trials evaluating the use of non-carbapenem β-lactam agents for ESBL infections are needed to resolve conflicting results generated by retrospective and observational studies, which likely reflect the presence of confounding/uncontrolled factors [5, 15]. Whenever equally efficacious alternate agents exist, consideration should be given to limit the use of carbapenems in the treatment of potentially less serious infection types (e.g. urinary tract infections) caused by ESBL-producing Enterobacterales, preserving carbapenems for the treatment of serious infections to prolong their effectiveness. Timely carbapenem de-escalation may also reduce the clinical use of these agents [15]. C/T has also shown promise as a non-carbapenem option [17]. Reanalysis of data from a randomized controlled Phase 3 clinical trial data [17] suggested that C/T may be an effective agent for treating patients with complicated urinary tract infection or complicated intraabdominal infection caused by ESBL-producing *E. coli* and *K. pneumoniae*, even when in vitro MICs for some patient isolates exceed the susceptible breakpoint of ≤2 μg/mL [17]. On the other hand, C/T is ineffective against isolates carrying carbapenemases, including class A (KPC) and class D (OXA) β-lactamases. It is inactive against metallo-β-lactamases (MBL), as are ceftazidime-avibactam, meropenem-vaborbactam, and imipenem/relebactam. The prevalence of all carbapenemases is low in the United States, but higher rates of serine carbapenemases and MBL have been reported in other geographic regions [27–30]. Local resistance patterns should be taken into account when making empiric therapy decisions.

MDR phenotypes are commonly observed among *P. aeruginosa* isolated from patients with lower respiratory tract infections in the United States [4, 6, 12, 13], including ICU patients [14]; ~ 15–30% of unique patient isolates of *P. aeruginosa* demonstrated an MDR phenotype in these studies. Although different definitions of multidrug-resistance complicate comparisons of MDR rates across studies, the MDR rate in the current study, 14.6% of lower respiratory tract infection isolates of *P. aeruginosa* tested from U.S. patients, was comparable to the aforementioned studies. Among these MDR *P. aeruginosa,* 76.2% of isolates were susceptible to C/T compared to < 20% susceptible to cefepime, ceftazidime, P/T, meropenem and levofloxacin (Table 2).

ICUs house vulnerable patients at high risk for infection. Gram-negative bacilli infecting patients in ICUs may be associated with elevated resistance rates and increased MDR [13]. In the current study, MDR rates were significantly higher and percent susceptible rates were significantly lower for P/T, cefepime, ceftazidime, and meropenem for ICU ward isolates compared to non-ICU ward isolates for *P. aeruginosa* (*P* < 0.05); no significant differences between ICU and non-ICU ward isolates were identified for any agent tested against Enterobacterales (*P* > 0.05). C/T was active against 92.9% of isolates of *P. aeruginosa* and Enterobacterales combined from ICU patients (Table 4).

Isolates of Gram-negative bacilli that are not susceptible to first-line agents (i.e., all categories of β-lactams, including carbapenems, and fluoroquinolones) are associated with increased therapeutic failure and mortality especially in severely ill patients, many of whom reside within ICUs [31, 32]. Aminoglycosides (e.g., amikacin) and polymyxins (e.g., colistin) have toxicities (aminoglycosides, ototoxicity and nephrotoxicity; polymyxins, nephrotoxicity) that limit their use for serious Gram-negative infections and these agents are often used in combination with other antimicrobial agents [11, 31]. These agents also have suboptimal pharmacokinetics (e.g., poor lung penetration), a narrow therapeutic index and have demonstrated inferior efficacy to β-lactams as first-line therapies [11]. As of 2020, isolates of *P. aeruginosa*, Enterobacterales, and *Acinetobacter* can no longer test as susceptible in vitro to colistin using 2020 CLSI M100 MIC breakpoints [33] following review of clinical and pharmacokinetic/pharmacodynamic data demonstrating limited clinical efficacy of colistin. β-lactams are the safest class of antimicrobial agents available to treat patients with serious infection caused by Gram-negative bacilli.

## Conclusions

We conclude that C/T remains highly active against isolates of *P. aeruginosa* infecting patients in the United States, including isolates with MDR phenotypes. C/T is an important treatment option for patients infected with *P. aeruginosa* isolates displaying an MDR phenotype that are not susceptible to other available β-lactam and fluoroquinolone anti-pseudomonal agents. Furthermore, C/T can be a potential alternative for patients where colistin or an aminoglycoside is a therapeutic consideration, given the well-established toxicities associated with those agents and their poor lung penetration. C/T was the most active β-lactam tested against *P. aeruginosa*. C/T had in vitro activity similar to cefepime and P/T against isolates of Enterobacterales, including putative ESBL-producing isolates of *E. coli* and *K. pneumoniae*. C/T was active against 93.5% of isolates of *P. aeruginosa* and Enterobacterales combined, which accounted for > 85% of all Gram-negative pathogens collected in the current study. Continuous surveillance monitoring for resistance is essential and provides critical data to guide empirical therapy, monitor the effectiveness of new antimicrobial agents and the emergence of resistance, and to monitor the performance of antimicrobial stewardship programs. Data from the current study support the use of C/T as an important treatment option for lower respiratory tract infections caused by MDR *P. aeruginosa* and Enterobacterales.

## Methods

### Bacterial isolates

In 2018 and 2019, 24 clinical laboratories in 16 states in the United States participated in the Study for Monitoring Antimicrobial Resistance Trends (SMART) global surveillance program and were each asked to collect up to 100 clinically significant, consecutive, aerobic or facultatively anaerobic Gram-negative bacilli per year from patients with lower respiratory tract infection. Isolates were restricted to one isolate per patient per Gram-negative species per year. In total, 3717 isolates of Gram-negative bacilli were collected (Additional file 3: Fig. S1). The five most common species were *P. aeruginosa* (33.3%), *K. pneumoniae* (11.6%), *E. coli* (9.7%), *Stenotrophomonas maltophilia* (7.3%), and *Serratia marcescens* (7.0%). The age distribution of patients from which isolates were collected was 0–17 years (*n* = 468, 12.6%), 18–64 years (*n* = 1781, 47.9%), ≥65 years (1363, 36.7%), and unknown age (105, 2.8%). *P. aeruginosa* (*n* = 1237) and Enterobacterales (*n* = 1938) together accounted for 85.4% (3175/3717) of all isolates collected. Among *P. aeruginosa* and Enterobacterales, 50.5% of isolates (1604/3175) were collected ≥48 h after the patient was admitted to the hospital, 34.8% (1104/3175) < 48 h post-admission, and for 14.7% (467/3175) the length of stay was not specified. All isolates were transported to a central laboratory (IHMA, Schaumburg, IL, USA) where they were re-identified using matrix-assisted laser desorption ionization-time of flight (MALDI-TOF) mass spectrometry (Bruker Daltonics, Billerica, MA, USA).

Ethical approval and informed consent was not required because all isolates received into the study followed multiple subcultures and were completely de-identified. The secondary research use of de-identified isolates is considered exempt research according to the Regulations for the Protection of Human Subjects in Research of the U.S. Department of Health and Human Services, Office for Human Research Protections (45 CFR 46).

### Antimicrobial susceptibility testing

MICs were determined using the CLSI (Clinical and Laboratory Standards Institute) reference broth microdilution method with frozen broth microdilution panels prepared at IHMA [33, 34]. MICs were interpreted using 2020 CLSI breakpoints [33]. MDR isolates were defined, phenotypically, as those testing resistant to three or more of the following eight sentinel antimicrobial agents: amikacin, aztreonam, cefepime, ceftazidime (Enterobacterales only), colistin, imipenem, levofloxacin, and P/T.

### Statistical analysis

The Chi-square statistic with Yates correction (XLSTAT version 2019.1.3) was used to establish statistical significance (*P* < 0.05) between variables.

## Supplementary Information

**Additional file 1: Table S1.** Species distribution of C/T-nonsusceptible Enterobacterales – SMART 2018–2019, United States

**Additional file 2: Table S2.** In vitro susceptibility to C/T of the most common MDR phenotypes of *P. aeruginosa* and Enterobacterales

**Additional file 3: Fig. S1.** Species distribution of 3717 isolates of Gram-negative bacilli collected from patients with lower respiratory tract infections – SMART 2018–2019, United States

## Data Availability

Datasets used and analyzed for this study are available from the corresponding author upon reasonable request.
