# Supplementary material for: Activity of ceftolozane/tazobactam against Gram-negative isolates from patients with lower respiratory tract infections – SMART United States 2018–2019
Source: BMC Microbiol. 2021 Mar 6;21:74. doi: 10.1186/s12866-021-02135-z (PMC7936229; doi:10.1186/s12866-021-02135-z)
Supplement: Supplementary file 1 — Additional file 1: Table S1. Species distribution of C/T-nonsusceptible Enterobacterales – SMART 2018–2019, United States [file 12866_2021_2135_MOESM1_ESM.pdf]

Additional file 1

Activity of Ceftolozane/Tazobactam against Gram-Negative Isolates from Patients with Lower Respiratory Tract Infections – SMART United States 2018-2019

James A. Karlowsky[1,2], Sibylle H. Lob[1*], Katherine Young[3], Mary R. Motyl[3] and Daniel F. Sahm[1]

[1] IHMA, 2122 Palmer Drive, Schaumburg, IL, 60173, USA
[2] Department of Medical Microbiology and Infectious Diseases, Max Rady College of Medicine, University of Manitoba, Winnipeg, MB, R3E 0J9, Canada
[3] Merck & Co., Inc., Kenilworth, NJ 07033, USA
* Correspondence: shlob@ihma.com

Table S1 Species distribution of C/T[a]-nonsusceptible Enterobacterales – SMART 2018-2019, United States

Species of Enterobacterales	*n*	% of all C/T-nonsusceptible Enterobacterales
Enterobacter cloacae	39	24.7
Klebsiella pneumoniae	32	20.3
Klebsiella aerogenes	31	19.6
Serratia marcescens	11	7.0
Escherichia coli	10	6.3
Citrobacter freundii	6	3.8
Enterobacter asburiae	5	3.2
Enterobacter cloacae complex	4	2.5
Klebsiella oxytoca	3	1.9
Citrobacter braakii	3	1.9
Enterobacter, non-speciated	2	1.3
Providencia stuartii	2	1.3
Enterobacter kobei	2	1.3
Enterobacter xiangfangensis	2	1.3
Raoultella planticola	1	0.6
Serratia ureilytica	1	0.6
Citrobacter amalonaticus	1	0.6
Enterobacter bugandensis	1	0.6
Proteus mirabilis	1	0.6
Raoultella ornithinolytica	1	0.6
Total	158[b]	100

[a] Abbreviation: C/T, ceftolozane/tazobactam
[b] 110/158 (69.6%) of isolates are intrinsic AmpC producers. *Escherichia coli*, *Klebsiella oxytoca*, *Klebsiella pneumoniae*, *Proteus mirabilis*, *Raoultella ornithinolytica* and *Raoultella planticola* are not intrinsic AmpC producers
