# Supplementary material for: Activity of ceftolozane/tazobactam against Gram-negative isolates from patients with lower respiratory tract infections – SMART United States 2018–2019
Source: BMC Microbiol. 2021 Mar 6;21:74. doi: 10.1186/s12866-021-02135-z (PMC7936229; doi:10.1186/s12866-021-02135-z)
Supplement: Supplementary file 2 — Additional file 2: Table S2. In vitro susceptibility to C/T of the most common MDR phenotypes of P. aeruginosa and Enterobacterales [file 12866_2021_2135_MOESM2_ESM.pdf]

Additional file 2

Activity of Ceftolozane/Tazobactam against Gram-Negative Isolates from Patients with Lower Respiratory Tract Infections – SMART United States 2018-2019

James A. Karlowsky[1,2], Sibylle H. Lob[1*], Katherine Young[3], Mary R. Motyl[3] and Daniel F. Sahm[1]

[1] IHMA, 2122 Palmer Drive, Schaumburg, IL, 60173, USA
[2] Department of Medical Microbiology and Infectious Diseases, Max Rady College of Medicine, University of Manitoba, Winnipeg, MB, R3E 0J9, Canada
[3] Merck & Co., Inc., Kenilworth, NJ 07033, USA
* Correspondence: shlob@ihma.com

Table S2 *In vitro* susceptibility to C/T of the most common MDR phenotypes of *P. aeruginosa*[a] and Enterobacterales[b]

Bacterial group/MDR phenotype[c]	*n* (% of all MDR isolates)[d]	% of isolates with each specific MDR phenotype that were susceptible to ceftolozane/tazobactam
P. aeruginosa		
aztreonam, imipenem, levofloxacin	31 (17.1)	96.8
aztreonam, cefepime, P/T, imipenem, levofloxacin	26 (14.4)	76.9
aztreonam, P/T, imipenem, levofloxacin	18 (9.9)	83.3
aztreonam, cefepime, P/T	17 (9.4)	88.2
aztreonam, cefepime, P/T, imipenem	13 (7.2)	76.9
Total for 5 most common MDR phenotypes among *P. aeruginosa*	105 (58.0)	85.7
Enterobacterales		
aztreonam, cefepime, ceftazidime, levofloxacin	52 (21.8)	90.4
aztreonam, ceftazidime, P/T	40 (16.8)	0
aztreonam, cefepime, ceftazidime	23 (9.7)	87.0
imipenem, levofloxacin, colistin	17 (7.1)	100
aztreonam, cefepime, ceftazidime, P/T, imipenem, levofloxacin	12 (5.0)	0
Total for 5 most common MDR phenotypes among Enterobacterales	144 (60.5)	58.3

[a] Sentinel antimicrobial agents used to define MDR for *P. aeruginosa* were aztreonam, cefepime, P/T (piperacillin/tazobactam), imipenem, levofloxacin, amikacin, and colistin.

[b] Sentinel antimicrobial agents used to define MDR for Enterobacterales were aztreonam, cefepime, ceftazidime, P/T, imipenem, levofloxacin, amikacin, and colistin.

[c] Sentinel agents listed as part of an MDR phenotype tested as resistant; sentinel agents not shown as part of an MDR phenotype tested as susceptible or intermediate.

[d] In total, there were 181 isolates of *P. aeruginosa* and 238 isolates of Enterobacterales with an MDR phenotype. The top 5 MDR phenotypes accounted for 58.0% (105/181) of all MDR isolates of *P. aeruginosa* and for 60.5% (144/238) of all MDR isolates of Enterobacterales.
