# Supplementary material for: Activity of ceftolozane/tazobactam against Gram-negative isolates from patients with lower respiratory tract infections – SMART United States 2018–2019
Source: BMC Microbiol. 2021 Mar 6;21:74. doi: 10.1186/s12866-021-02135-z (PMC7936229; doi:10.1186/s12866-021-02135-z)
Supplement: Supplementary file 3 — Additional file 3: Fig. S1. Species distribution of 3717 isolates of Gram-negative bacilli collected from patients with lower respiratory tract infections – SMART 2018–2019, United States [file 12866_2021_2135_MOESM3_ESM.pdf]

Additional file 3

Activity of Ceftolozane/Tazobactam against Gram-Negative Isolates from Patients with Lower Respiratory Tract Infections – SMART United States 2018-2019

James A. Karlowsky[1,2], Sibylle H. Lob[1*], Katherine Young[3], Mary R. Motyl[3] and Daniel F. Sahm[1]

[1] IHMA, 2122 Palmer Drive, Schaumburg, IL, 60173, USA
[2] Department of Medical Microbiology and Infectious Diseases, Max Rady College of Medicine, University of Manitoba, Winnipeg, MB, R3E 0J9, Canada
[3] Merck & Co., Inc., Kenilworth, NJ 07033, USA
* Correspondence: shlob@ihma.com

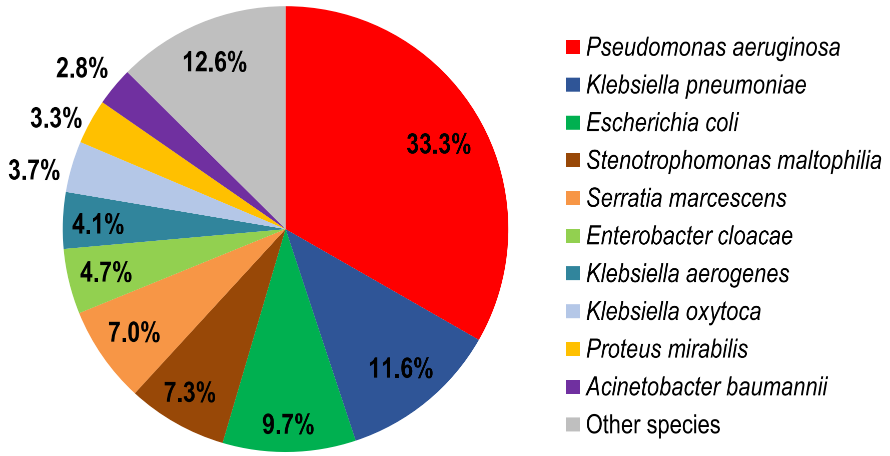

Figure S1 Species distribution of 3717 isolates of Gram-negative bacilli[a] collected from patients with lower respiratory tract infections – SMART 2018-2019, United States

[a] *Pseudomonas aeruginosa* and Enterobacterales accounted for 85.4% (3175/3717) of all isolates of Gram-negative bacilli collected by the SMART surveillance program in the United States in 2018-2019
